# A 0.18-μm BCD Adaptive-On-Time Buck DC-DC Converter with 96.03% Peak Efficiency and 100% Duty Cycle

**DOI:** 10.3390/mi17030279

**Published:** 2026-02-25

**Authors:** Tianwen Li, Shoubin Qi, Hongjin Liu

**Affiliations:** 1 Beijing Aerospace Shenzhou Intelligent Equipment Technology Co., Ltd., Beijing 100086, China; litianwen@sunwisespace.com; 2 Beijing Sunwise Space Technology Ltd., Beijing 100190, China; 3 Beijing Institute of Space Science and Technology Information, Beijing 100086, China

**Keywords:** constant on time (COT), 100% duty cycle, buck DC-DC, high efficiency

## Abstract

This paper proposes a high-efficiency adaptive-on-time buck DC-DC converter (HEAOT–Buck) capable of supporting a 100% duty cycle. The converter operates over an input voltage range of 3–18 V and provides an adjustable output voltage from 0.9 to 5.5 V, delivering up to 1 A load current. Implemented using a 0.18 µm BCD process, the chip attains a peak efficiency of 96.03%. When the load current changes from 0.1 to 1 A, the output voltage regulation remains within 0.36%, while the line regulation below 0.55%. At a switching frequency of 2.5 MHz and a 0.5 A load condition, the output voltage ripple measures at approximately 22 mV. Under a load transient from 0 to 1 A, the output voltage settles within 10 µs.

## 1. Introduction

In the current era of digitalization and intelligence, electronic devices are becoming increasingly sophisticated, resulting in greater demands on power management systems. Effective power management solutions are essential for extending battery life, accommodating rapidly changing power requirements across diverse operating modes, and maintaining stable and reliable operation in complex environments. As a key component within a power management system, the step-down DC-DC converter plays a vital role in efficiently and stably converting the input voltage to the desired output voltage and delivering it accurately to the load [1,2]. However, conventional Buck converters exhibit notable limitations in various scenarios. Their efficiency significantly decreases under light-load or no-load conditions, dissipating excessive energy as heat [3,4], which is especially critical in portable devices and systems that require long-term stable operation. In lithium battery voltage conversion applications, the nominal voltage of a lithium battery is 3.7 V, and its typical operating voltage range spans 3 V–4.25 V. The battery voltage gradually decreases as its charge depletes; when the output voltage is configured to 3.3 V, the traditional buck converter topology cannot operate properly once the input voltage approaches 3.3 V. Thus, a buck converter topology supporting 100% of the duty cycle is required to address such application scenarios. During load transients, characterized by abrupt changes from light to heavy load, the output voltage often experiences severe fluctuations and requires considerable time to recover, potentially leading to device malfunctions or damage. Moreover, when entering a power-saving mode, converters frequently experience pronounced voltage sag and additional power consumption, while abrupt mode transitions can severely degrade device performance and user experience [5].

To address the drawbacks of traditional Buck converters and fulfill the stringent power management requirements of modern electronic systems, several Buck DC-DC control architectures have been developed [6]. These architectures enable precise regulation of output voltage and current by directly controlling the power switches, thereby significantly enhancing dynamic response speed and output voltage accuracy [7]. They can also seamlessly transition into power-saving modes under light-load or no-load conditions, effectively reducing power consumption and improving light-load efficiency [8,9]. In addition, they ensure rapid output stabilization during sudden load changes, maintaining reliable device operation. Most importantly, their mode transitions are exceptionally smooth, producing minimal disturbance in output voltage and power delivery, thus providing continuous and stable energy to the system [10,11,12].

This paper presents the design of a high-performance step-down DC-DC converter with 100% duty cycle based on the HEAOT–Buck architecture. By carefully designing key circuits, such as adaptive on-time control, zero-current detection (ZCD), and power saving mode switching modules, the proposed converter achieves high efficiency, fast transient response, and precise output voltage regulation across the entire load range, thereby significantly improving the overall performance of the power management system. This paper provides a detailed analysis of the converter’s system architecture and operating principles, discusses the circuit implementation of each functional module, and verifies its outstanding performance through experimental evaluation [13,14].

The rest of this paper is organized as follows. Section 2 introduces the system architecture of the proposed HEAOT–Buck converter. Section 3 describes the primary modules and their operating principles. Section 4 presents the experimental results and discussion, and Section 5 presents the conclusions.

## 2. HEAOT–Buck Architecture

**Figure 1:**
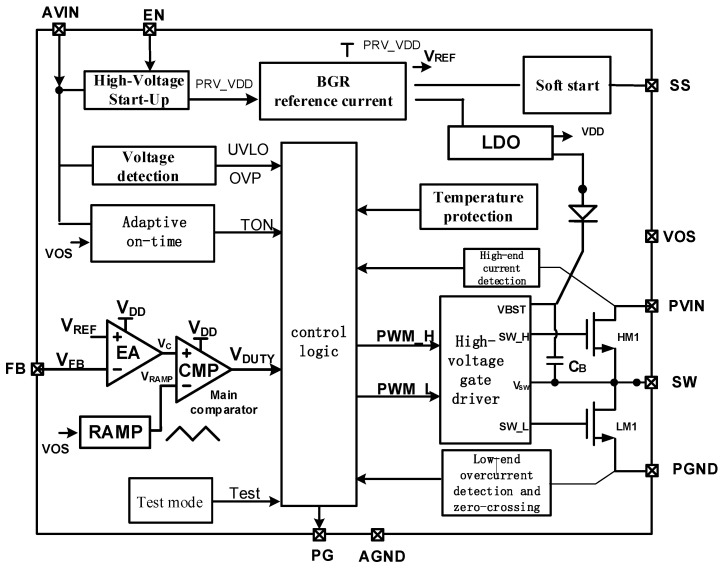
HEAOT–Buck Block Diagram.

Figure 1 illustrates the system block diagram of the proposed Buck DC-DC converter based on the HEAOT–Buck architecture. Operating at a typical switching frequency of up to 2 MHz, the converter supports the use of compact inductors. By employing a DCS control topology, the converter achieves a fast transient response and high output voltage accuracy. It provides a wide input voltage range from 3 to 18 V, making it suitable for systems powered by lithium-ion batteries, other types of rechargeable batteries, or 12 V intermediate power rails. The output voltage is adjustable from 0.9 to 5.5 V and supports a continuous output current of up to 1 A in 100% duty cycle mode. The soft-start (SS) pin regulates the ramp rate of the output voltage, enabling the converter to function as a standalone power supply or within voltage-tracking configurations. Through proper configuration of the enable (EN) pin and the open-drain power good (PG) pin, power sequencing can also be achieved. In power-saving mode, the converter typically consumes a quiescent current of approximately 35 µA at the input voltage *V*_IN_. Under light-load conditions, it automatically and smoothly transitions into power-saving operation, ensuring high efficiency across the entire load range. In shutdown mode, the current power consumption of the device remains below 2 µA.

**Figure 2:**
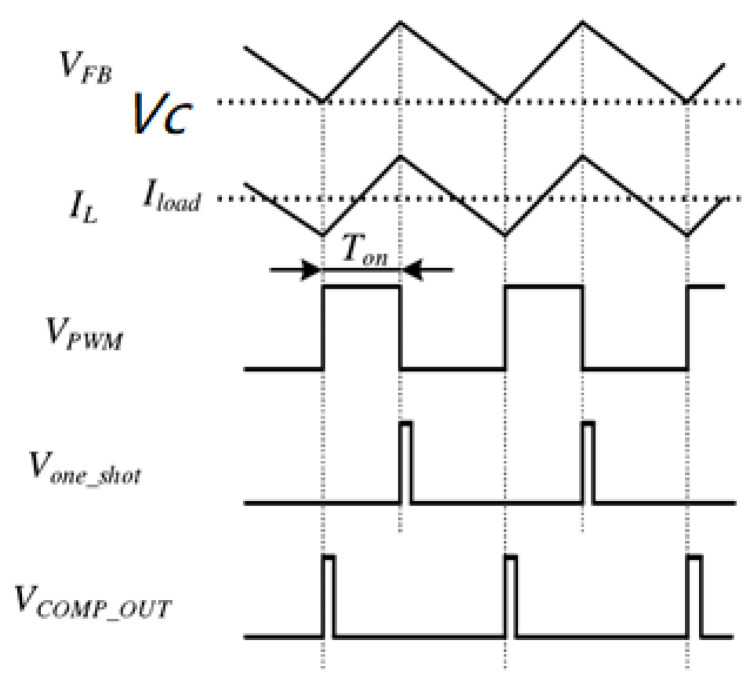
Control Mechanism of the Constant on Time (COT) Buck Converter.

As shown in Figure 2, the output voltage *V*_c_ of the operational amplifier represents the difference between the reference voltage *V*_ref_ and the feedback offset voltage *V*_os_. Owing to the amplifier’s gain, *V*_os_ stabilizes at *V*_ref_ × *K*, where *K* denotes the voltage division ratio. When the feedback voltage *V*_fb_ falls below *V*_c_, the comparator output *V*_comp_out_ switches to high, turning on the high-side switch (HS). Consequently, the inductor current rises linearly, while the output voltage ripple and the *V*_fb_ ripple increase, forcing *V*_comp_out_ to return to low, forming a narrow pulse. During the high state of *V*_comp_out_, the monostable timer is triggered and remains active for the fixed on-time *T*on. Its output *V*_one_shot_ then transitions to high, resetting the SR latch. Afterward, the low-side switch (LS) turns on, allowing the inductor current to decrease and the output voltage ripple to diminish until *V*_fb_ again drops below *V*_c_, restarting the control cycle.

To ensure a seamless transition from PWM to 100% duty cycle operation, the logic design incorporates a logical OR function between the PWM comparator output *V*_comp_out_ and the timer output on-time signal. In PWM mode, *V*_comp_out_ initially transitions high to turn on the HS. As the output voltage and *V*_fb_ ripples increase, *V*_comp_out_ quickly returns to low, forming a narrow pulse. Therefore, HS turn-off is primarily determined by the fixed on-time, since the pulse width of *V*_comp_out_ is shorter than *T_on_*. As the output voltage *V*_out_ approaches the input voltage *V_in_* and the duty cycle increases, the PWM comparator pulse gradually widens. When the pulse width exceeds *T_on_*, HS turn-off is instead governed by *V*_comp_out_, allowing the converter to operate in a voltage-mode-like control manner.

When *V*_out_ ≥ *V_in_*, the error amplifier (EA) functions as a comparator, causing the PWM comparator output to remain high, which keeps the HS switch continuously on. This mechanism allows the converter to seamlessly transition into 100% duty cycle operation. If the HS switch remains on for a period longer than the predefined duration, the system automatically enters a low-power mode and exits it once switching activity is detected again.

## 3. Circuit Implementation

The circuit implementation of the HEAOT–Buck comprises four main functional modules: the power management module, the control logic module, the power conversion module, and the protection and monitoring module.

### 3.1. Power Management Module

**Figure 3:**
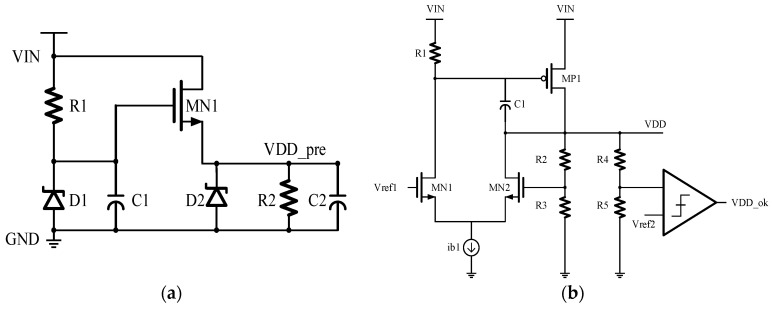
Power Management Module Schematic: (**a**) Pre-Regulation Circuit; (**b**) LDO.

To ensure the safe and stable operation of the HEAOT–Buck over a wide input voltage range of 3–18 V, an on-chip power management system is incorporated. This system consists of a pre-regulator circuit and a fast-transient low dropout regulator, as illustrated in Figure 3. The system first converts the high input voltage *V*_in_ into an intermediate voltage *V*_DDPRE_ using a source-follower-based pre-regulator. An integrated clamp diode limits and stabilizes *V*_DDPRE_ below 5.5 V, thereby reducing voltage stress on subsequent stages while supporting bootstrap startup functionality.

The fast transient low dropout module adopts a dual independent power supply structure, providing separate voltages for the power driver domain (*V*_DDDRV_) and the logic control domain (*V*_DD_LOGIC_). Each LDO incorporates internal RC compensation and voltage comparators that enable rapid response to load variations. In addition, the module outputs a *V*_DDOK_ signal to indicate stable power conditions.

This compact and functionally complementary design supports step-down startup operation, ensuring that the downstream modules are activated only after the power supply reaches the required operating levels. Simulation results confirm that across the full temperature range of −40 °C to +125 °C and under all process corners, V_DDPRE_ remains stable below 5.5 V. The LDO consistently outputs 5 V when the input voltage is within 5–18 V, maintaining a dropout voltage of less than 30 mV at 20 mA load. These results verify the module’s capability to suppress temperature-induced variations, ensure output voltage stability, and provide reliable startup performance.

### 3.2. Control Logic Module

**Figure 4:**
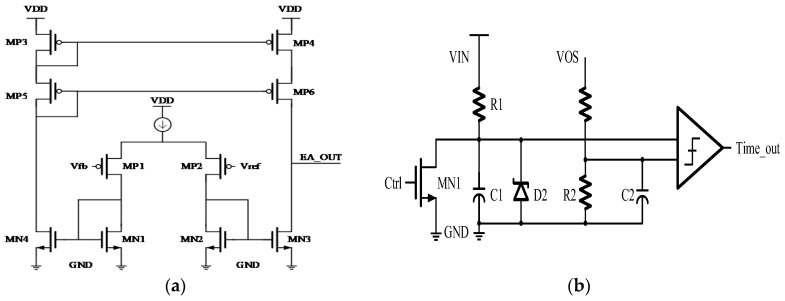
Control Logic Module Circuit Diagram: (**a**) Error Amplifier (EA); (**b**) Adaptive On-Time Generator (On-Timer).

The HEAOT–Buck utilizes a synchronous Buck control strategy based on constant on-time (COT). The control logic integrates several functional submodules, including an EA, a ramp generator, an on-time generator (On-Timer), SS control, and an on-chip oscillator, thereby forming a complete closed-loop control system, as shown in Figure 4.

The EA compares the feedback voltage with an internal reference voltage *V*_REF_ (0.8 V) and generates a regulation signal. This signal is then compared with a ramp waveform to determine the turn-on instant of the high-side power switch, effectively preventing subharmonic oscillation under high-duty-cycle conditions. The On-Timer establishes a COT using an on-chip RC network, with its output period adaptively adjusted according to the input to output voltage ratio to maintain a stable switching frequency.

During the power-up sequence, an external capacitor connected to the SS pin is charged by a constant current source, ensuring a smooth rise in the output voltage and preventing inrush current. When the SS voltage reaches *V*_REF_, the SS_DONE signal is generated to close the main feedback control loop. The on-chip oscillator provides the base clock for the system operation and can be routed to the test mode output for testing and debugging purposes.

This control system exhibits a compact architecture and fast dynamic response while integrating multiple functions, such as SS operation, oscillation suppression, and adaptive frequency regulation. Simulation results indicate that when the load current steps from 0.1 to 1 A, the output voltage returns to steady state within 10 µs without overshoot. During SS, the output voltage rises smoothly with a startup duration of approximately 1.5 ms. The system demonstrates stable operation across the full switching frequency range, showing excellent bandwidth, regulation accuracy, and frequency adaptability, thereby confirming the robustness and fast transient response of the closed-loop control system.

### 3.3. Power Conversion Module

**Figure 5:**
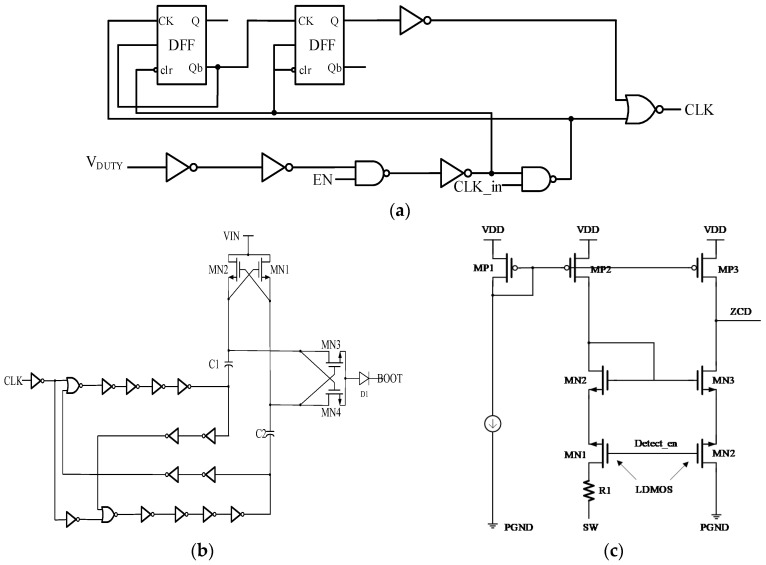
Circuit Diagram of the Power Conversion Module: (**a**) 100% Duty-Cycle Control Circuit; (**b**) Charge-Pump Driver Module; (**c**) Zero-Current Detection (ZCD).

The integrated power stage of the HEAOT–Buck adopts a synchronous Buck topology, as illustrated in Figure 5. It consists of five major submodules: high-side and low-side power MOSFETs, 100% duty-cycle control circuit, charge-pump driver, and ZCD circuit.

100% duty-cycle control: As shown in Figure 5a, if the V_DUTY_ remains high for consecutive cycles, the CLK is enabled, the charge-pump driver module is activated, and the chip automatically enters the 100% duty cycle mode; when the V_DUTY_ is low, the chip exits the 100% duty cycle mode.

Power switches: Both the HS and LS employ 20 V LDMOS transistors, featuring on-resistances below 90 and 40 mΩ, respectively. This design minimizes conduction losses and ensures efficient operation even under high-frequency switching conditions.

Charge-pump driver: As shown in Figure 5b, the on-chip charge pump utilizes a capacitor-doubling configuration to boost the bootstrap voltage to a level exceeding *V*_IN_ + 5 V. This guarantees reliable turn-on of the high-side transistor, even during 100% duty cycle operation.

ZCD: As shown in Figure 5c, the zero-crossing of the inductor current is detected by comparing the SW node potential with 0 V. Under light-load conditions, the synchronous rectifier is promptly turned off to avoid reverse-current losses.

### 3.4. Protection and Monitoring Module

**Figure 6:**
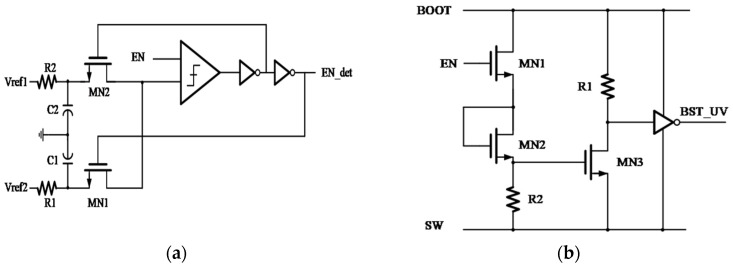
Protection and Monitoring Module Circuit Diagrams: (**a**) EN_DET; (**b**) BST_UV.

To improve system safety and debuggability under complex operating conditions, the DCS chip integrates power-on qualification, I/O voltage monitoring, power-domain protection, and test-observation functions within its protection and monitoring module (Figure 6), forming a highly coordinated on-die protection architecture.

The EN_DET block adopts a Schmitt–Trigger structure to sense the EN pin and, together with on-chip delay logic, ensures that downstream modules are activated only after the enable signal becomes stable. A power-on-reset circuit continuously monitors *V*_DD_PRE_ with respect to a band-gap reference; it holds the system in reset state when *V*_DD_PRE_ is below 3 V, preventing false start-up. During normal operation, the DET block compares *V*_IN_ with the FB voltage in real time; when an under-voltage (V_IN_UV_/F_B_UV_) or over-temperature (OTP) event is detected, the PG block immediately drives the PG pin high to indicate a fault and initiates protective shutdown. The BST_UV detection circuit compares the BST-SW voltage with a 2.95 V threshold; if the voltage falls below this level, the high-side FET is disabled to prevent damage caused by insufficient gate drive.

All protection thresholds maintain minimal variation over the full temperature range (−40 °C to 150 °C) and across all process corners (FF/TT/SS), demonstrating high consistency. Compact, fast-responding, and logically organized, this protection module serves as a key component for achieving high reliability and testability in the HEAOT–Buck system.

### 3.5. System Stability of HEAOT–Buck

**Figure 7:**
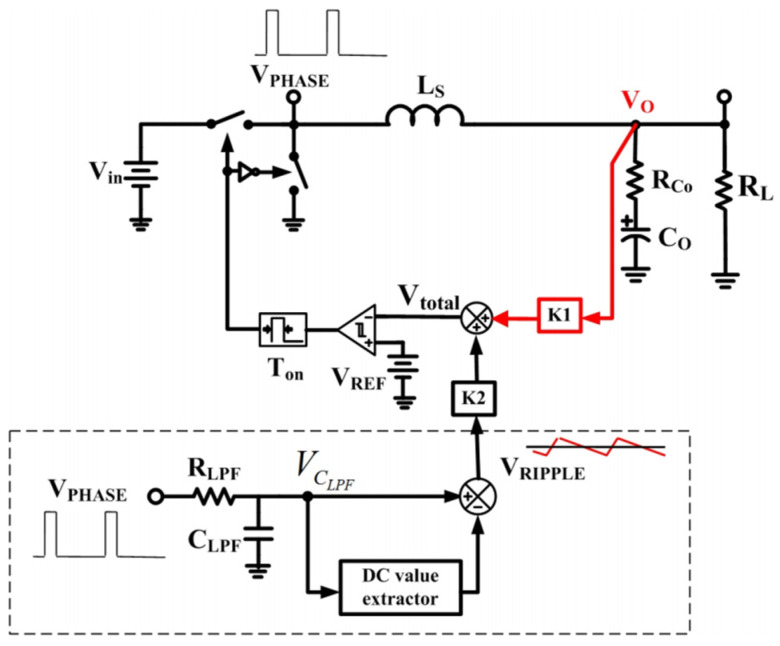
Block diagram of the COT control with a virtual inductor current ripple generator [15].

COT control scheme often encounters instability issue of subharmonic oscillation when ceramic capacitors are used for converter output filter capacitors. The on-chip ripple compensation circuit in this paper is designed with reference to [15,16]. As shown in Figure 7, virtual inductor current (VIC) ripple is used to alleviate this problem. The novel control scheme improves system stability of COT control without adding extra components in IC implementation. Based on Padé approximation [15], the transfer function from control to output can be derived as (1).

(1) vo(s)vc(s)≈11+s⋅Ton2+s2⋅Ton2π2⋅1+s⋅RcoCo1+s⋅RcoCo1+Ls⋅kRco⋅τLPF−Ton2+s2⋅Ts2π2

where *T_on_* is the on time of high side driver, *R_CO_* is the ESR resistor of the output capacitor, *C_O_* is the output capacitor, *Ls* is the inductor, *k* = K2/K1, and *τ_LPF_* = R_LPF_ × C_LP_F.

It can be seen that (1) has complex poles which may go into the right-half plane and cause system instability if the coefficient of s is negative. The above criterion is used to determine the stability criteria of the proposed control as shown in (2).

(2) $$R_{co}C_{o}\left(1+\frac{L_{s}\cdot k}{R_{co}\cdot \tau_{\mathrm{LPF}}}\right)-\left(\frac{T_{on}}{2}\right)\geq 0$$

As long as the actual operating conditions of the chip are taken into account during the design process and a certain design margin is reserved, it can be ensured that the chip operates stably at all times without subharmonic oscillation.

## 4. Chip Test Result

**Figure 8:**
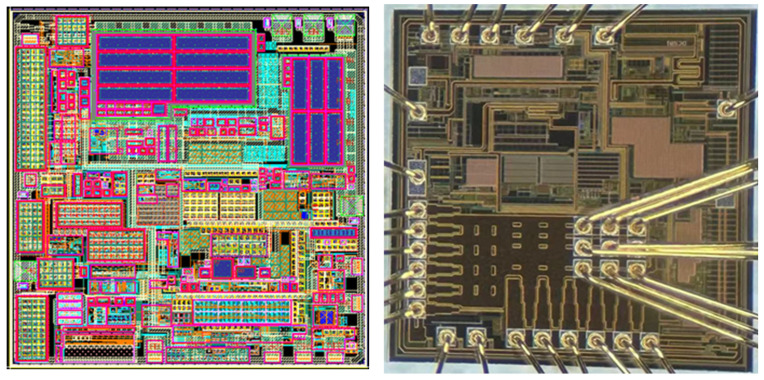
Layout and Chip Diagram.

The proposed HEAOT–Buck converter was fabricated using a 0.18-μm BCD 1P4M process, occupying a die area of 2.1 mm × 2.1 mm, as shown in Figure 8. The power stage adopts deep-N-well isolation. The high-side MOSFET exhibits an on-resistance of 85 mΩ, while the low-side MOSFET exhibits an on-resistance of 45 mΩ, supporting a maximum absolute input voltage of 20 V.

**Figure 9:**
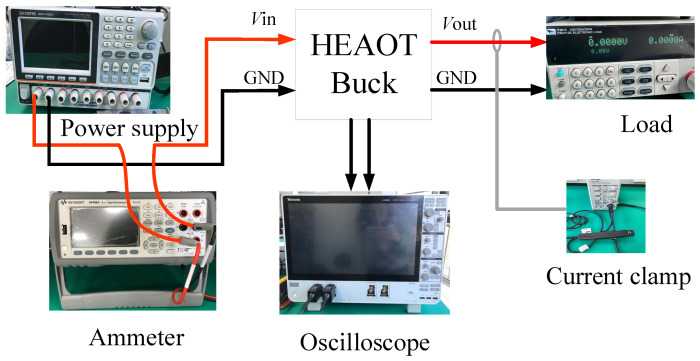
Block Diagram of the Test System.

**Figure 10:**
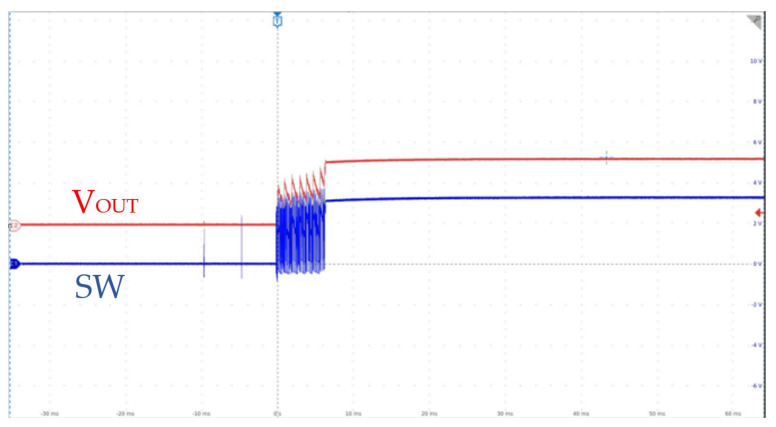
100% Duty-Cycle Mode Test.

The chip test setup is illustrated in Figure 9. Measurement results show that the EN threshold is 1.6 V (rising)/0.9 V (falling). The SS slope is defined by the external capacitor connected to the SS/TR pin; with *C*_SS_ = 0.033 µF, the measured start-up time is 16.5 ms, which matches the theoretical *trise* = *C*_SS_ × 0.8 V/2.5 µA within 98%. When the output voltage is configured as 3.3 V through the output divider resistor, and the input voltage is only 3.29 V (which is lower than the configured output voltage), the chip will automatically enter the 100% duty cycle mode, as shown in Figure 10.

**Figure 11:**
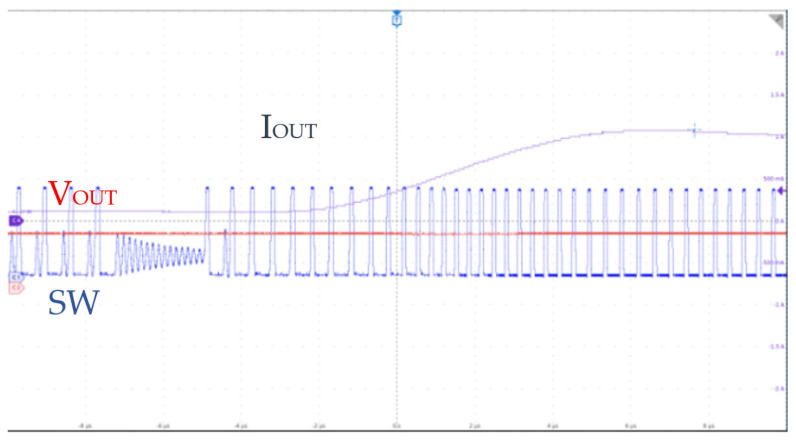
Output Waveform Test Under Load Step from 0.1 to 1 A.

Experimental verification confirms that the HEAOT–Buck control scheme provides a superior transient response. As shown in Figure 11, when the load current increases from 0.1 to 1 A, the voltage settles within 3 µs.

The output-voltage ripple exhibits strong dependence on the switching frequency. Under *V_IN_* = 18 V and *I*_OUT_ = 0.1 A, the ripple measures 84.98 mVp-p at *f*_SW_ = 1.25 MHz and decreases to 25.74 mVp-p at 2.5 MHz, consistent with the theoretical *V*_ripple_ ∝ 1/*f*_SW_ relationship. The converter also demonstrates excellent thermal stability across –40 °C to 125 °C: load regulation is 0.36%, line regulation is 0.39%, and the total output-voltage deviation remains within ±1.5%. These results confirm the HEAOT–Buck architecture’s advantage in achieving high dynamic performance, low ripple, and stable operation across a wide temperature range.

**Figure 12:**
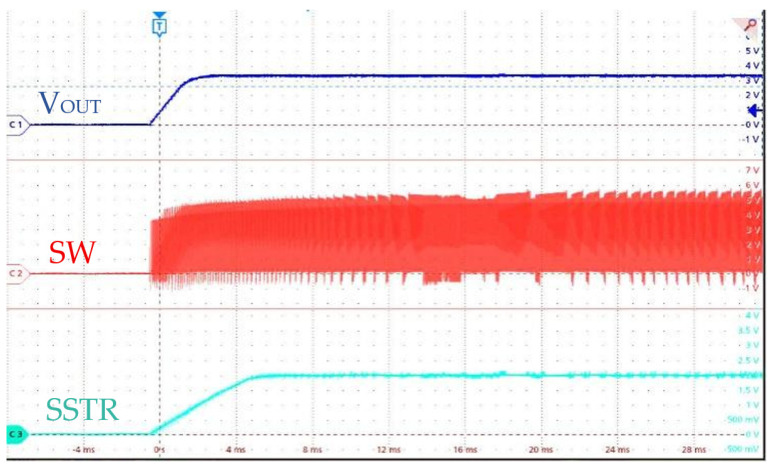
Waveforms of SSTR, SW, and V_OUT_ During Chip Startup.

Figure 12 shows the startup waveforms of the SS (SSTR), switching node (SW), and output voltage (V_OUT_). The chip initiates with a smooth and well-controlled SS ramp, effectively preventing inrush current and output overshoot. The SW node exhibits clean switching transitions, reflecting stable converter behavior during startup.

**Figure 13:**
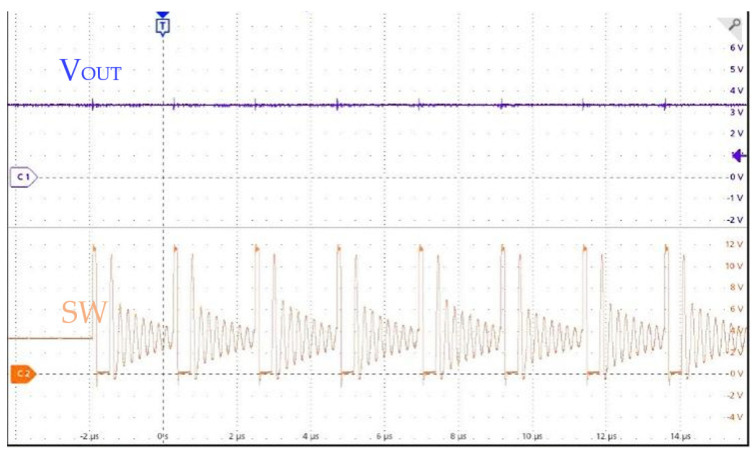
No-load: SW and V_OUT_ Waveforms.

**Figure 14:**
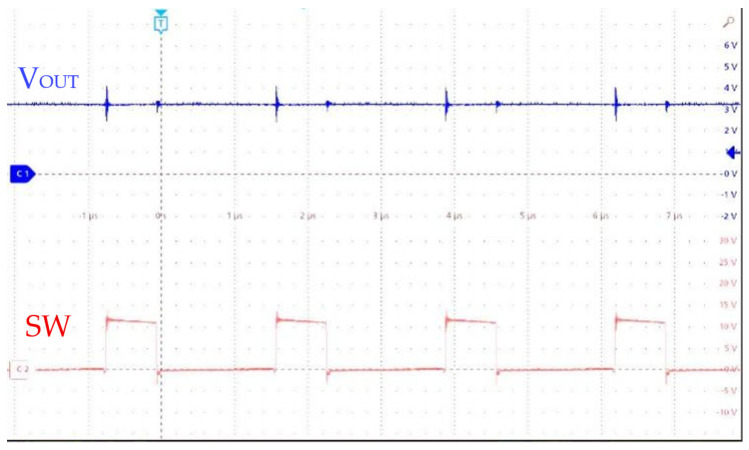
1 A load: SW and V_OUT_ Waveforms.

The steady-state behavior of the converter was evaluated under both no-load and 1 A load conditions at a 12 V input. As shown in Figure 13 and Figure 14, the SW node maintains stable switching characteristics, and the output voltage remains well-regulated. The measured output-voltage ripple stays within the specified limits, demonstrating the robustness of the feedback control loop and the effectiveness of the output filter network.

**Figure 15:**
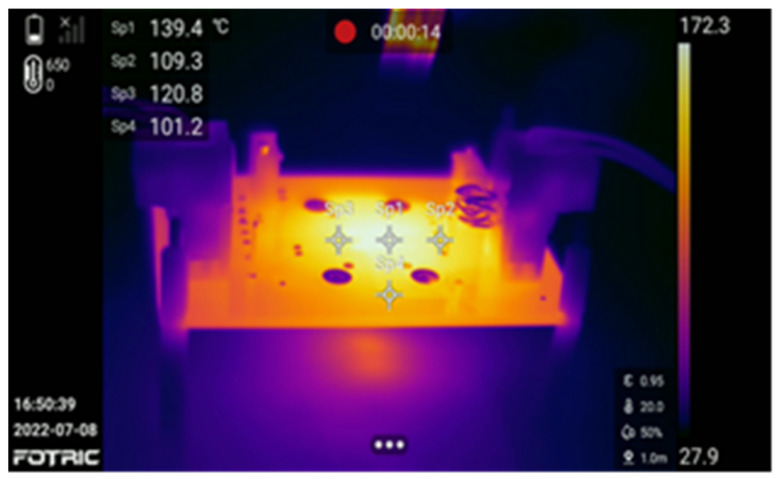
Over-Temperature Protection Test.

The chip was comprehensively characterized across a wide load range. As summarized in Table 1, when *I*_OUT_ < 0.2 A, the HEAOT–Buck control loop automatically transitions into PSM, reducing the switching frequency *f*_SW_ to 200 kHz. At a light-load current of 10 mA, the efficiency remains at 84.6%, consistent with the result reported in Ref. [17]. Under *V*_IN_ = 5 V and *I*_OUT_ = 0.1 A, the current-reuse sensing scheme effectively minimizes conduction losses, enabling the converter to achieve a peak efficiency of 96.03%. The shutdown quiescent current measures only 1.45 µA at *V*_IN_ = 12 V, which significantly extends battery operating life. Thermal-management characterization, shown in Figure 15, indicates that the junction temperature rises to 151 °C at full load before the protection mechanism activates. A hysteresis window of 10 °C is incorporated to prevent thermal oscillation and ensure stable recovery.

As summarized in Table 2, the proposed HEAOT–Buck topology demonstrates comprehensive advantages in terms of input/output voltage range, integration level, and power conversion efficiency. It supports the widest input range of 3–18 V, thereby eliminating the need for pre-regulation and accommodating common system buses such as 5 V, 9 V, 12 V, and 15 V. Meanwhile, the continuous output range of 0.9–5.5 V enables versatile rail generation, covering 1 V core, 1.8 V I/O, 3.3V analog and 5 V motor, significantly reducing the number of on-board power supplies. The converter achieves a peak efficiency of 96.03%, the highest among the five compared candidates, which effectively minimizes energy loss and prolongs battery lifetime. Only this work supports 100% duty cycle. Moreover, the maximum switching frequency of 2.5 MHz enables the use of a smaller inductor while maintaining high conversion efficiency. Overall, the proposed design represents a fully integrated, high-performance power converter, ideally suited for area-constrained applications such as IoT devices, wearable electronics, and micro-module power systems.

## 5. Conclusions

This paper presents a HEAOT–Buck converter developed for IoT and portable electronic applications, providing high efficiency and rapid transient response. The chip achieves a peak efficiency of 96.03% at *V*_IN_ = 5 V and *I*_OUT_ = 0.1 A and maintains 84.63% efficiency at *V*_IN_ = 12 V and *I*_OUT_ = 1 A, outperforming comparable COT architectures. It operates from a 3 to 18 V input range and delivers an adjustable 0.9–5.5 V output, maintaining voltage deviation within ±1.5% across the full –40 °C to 125 °C temperature range. The quiescent current is only 35 µA, and the shutdown current is below 2 µA, significantly enhancing battery endurance. With these characteristics, the converter is highly suitable for IoT end-nodes, automotive infotainment systems, industrial sensors, wearable devices, and other energy-sensitive applications.

## Figures and Tables

**Table 1:** Measured efficiency at *f*_SW_ = 2.5 MHz, *V*_OUT_ = 3.3 V.

*V*_in_ (V)	Load/A	*I*_in/_A	*V*_out/_V	Efficiency (%)
5	0.1	0.0690	3.313	96.03
	0.2	0.1389	3.314	95.44
	0.5	0.3561	3.314	93.06
	0.8	0.5747	3.312	92.21
	1	0.7315	3.311	90.53
12	0.1	0.0309	3.317	89.46
	0.2	0.0646	3.321	85.68
	0.5	0.1631	3.325	84.94
	0.8	0.2596	3.326	85.41
	1	0.3278	3.329	84.63

**Table 2:** Performance Comparison of Chips.

Design	Input Range (V)	Output Range (V)	Load Range (A)	Peak Efficiency (%)	Frequency (MHz)	100% Duty Cycle	Process (μm)
This work	3–18	0.9–5.5	0–1	96.03	2.5	Yes	0.18
RBCOT + Current Sensing [18]	3.3	0.9–1.8	0–1	92.32	1	No	0.18
DCCRICOT Buck [19]	3.3	1.1	0.01–1.1	94.9	5	No	0.18
Dual-Modulation AOT Buck [20]	5.5–15	0.5–5	0–5	85.14	2.17	No	0.18
PWM/PFM IoT [8]	3.6–5.4	3.3	0–0.1	95.70	1	No	0.18

## Data Availability

The original contributions presented in this study are included in the article. Further inquiries can be directed to the corresponding author.
